# Changes in Beck Depression Inventory scores in prostate cancer patients undergoing androgen deprivation therapy or prostatectomy

**DOI:** 10.1371/journal.pone.0234264

**Published:** 2020-06-15

**Authors:** Dongseong Shin, Sung Ryul Shim, Chang Hee Kim

**Affiliations:** 1 Department of Pharmacology, Gachon University College of Medicine, Incheon, Korea; 2 Clinical Trials Center, Gachon University Gil Medical Center, Incheon, Korea; 3 Department of Preventive Medicine, Korea University College of Medicine, Seoul, Republic of Korea; 4 Department of Urology, Gil Medical Center, Gachon University College of Medicine, Incheon, Republic of Korea; National Health Research Institutes, TAIWAN

## Abstract

**Objectives:**

Androgen deprivation therapy (ADT) has seen increasing use as a prostate cancer treatment in recent years and has proven medically effective in numerous contexts. The treatment, however, is associated with a host of side effects including depression. Managing the psychological wellbeing of prostate cancer patients is important for maximizing their survival outcomes. Thus, this study aimed to evaluate depressive symptomatology in patients with androgen deprivation therapy (ADT) compared with that in patients who underwent prostatectomy and to explore the factors that affect depressive symptoms, which might occur during ADT.

**Methods:**

One hundred and seven patients undergoing ADT (ADT group) and prostatectomy (Operation group) were enrolled. Adjustments were made for differences in characteristics between groups using a propensity score model with stabilized weights before treatment. Depressive symptoms between groups were compared using the Beck Depression Inventory (BDI) before treatment and six months after treatment initiation. To identify factors affecting depressive symptoms during ADT, multivariate regression analysis was performed on the mean change in BDI score, age, body mass index, testosterone level, prostate-specific antigen level, the international index of erectile function (IIEF), and the Gleason score.

**Results:**

The BDI score significantly increased in the ADT group compared to the operation group six months after treatment initiation (*p* < 0.001). Multivariate regression analysis revealed that before ADT, the BDI score was higher by 0.446 according to the IIEF. During ADT, the BDI score increased by 1.579 according to changes in BMI (*p* = 0.021) and decreased by 0.01 according to changes in testosterone levels (*p* = 0.034).

**Conclusion:**

Depressive symptoms can be exacerbated in prostate cancer patients undergoing ADT. Efforts are needed to diagnose and treat depression appropriately, especially if depressive symptoms change in ADT patients with a high IIEF score before ADT, or reduced testosterone levels or increased BMI during ADT.

## Introduction

Prostate cancer is the most common cancer in men, with around 240,000 new patients being diagnosed in the United States every year [1]. Prostate cancer is generally treated with prostatectomy, radiation therapy, brachytherapy, and androgen-deprivation therapy (ADT). The number of patients undergoing ADT is recently on the rise [2], as it is recommended for patients with an elevation of prostate-specific antigen (PSA) following prostatectomy and radiation therapy, patients who are asymptomatic for metastasis and have node-positive status, and patients whose metastasis has been radiologically confirmed but are asymptomatic [3]. However, ADT is known to have side effects such as loss of libido, gynecomastia, erectile dysfunction, anemia, cardiovascular disease, osteoporosis, and depression. Specifically, depression has been shown to be associated with ADT [4]. Recently, maintaining a good quality of life (QoL) for prostate cancer patients undergoing ADT has become particularly important because the survival of these patients has increased [5]; thus, managing psychological problems, particularly depressive symptoms, is considered to be critical [6]. However, due to the common misperception that the elderly are less likely than younger people to experience psychological pain when facing cancer, studies evaluating the psychological problems associated with prostate cancer undergoing ADT have been rare [7].

Thus, the objective of this study was to evaluate depressive symptomatology in patients with ADT compared with that in patients who underwent prostatectomy. Further, we explored the factors that affect depressive symptoms, which might occur during ADT, with the aim of enhancing the QoL of prostate cancer patients undergoing ADT using appropriate diagnosis and management.

## Patients and methods

### Participant recruitment

This study is an exploratory study to evaluate depressive symptomatology in patients with ADT compared with that in patients who underwent prostatectomy and to explore the factors that affect depressive symptoms. Thus, this study was conducted on 107 patients who met the following criteria: Patients who were diagnosed with prostate cancer and were scheduled to undergo ADT or prostatectomy at the Gil Medical Center between September 2017 and August 2018 were enrolled in this study. Patients who were to undergo ADT were assigned to the ADT group and those who were to undergo prostatectomy were assigned to the operation group. Written informed consent forms were obtained before enrollment. The inclusion criteria were as follows: (1) Patients undergoing ADT for at least six months (ADT group) according to the planned schedule, and patients who were available for at least six months of follow-up after prostatectomy (operation group), (2) patients capable of reading, understanding, and completing the questionnaire on their own, (3) patients who had an elementary school education or higher, and (4) patients who were married. The exclusion criteria were as follows: (1) patients who had a history of psychiatric disorders, including depression and neurological disorders involving the brain, (2) patients who had been diagnosed with a different type of cancer or had been treated for a different type of cancer prior to participation in this study, and (3) patients who had undergone a procedure that affects male hormones, such as testosterone supplementation, ADT, and anti-androgen, prior to participation in this study.

The principles of the Helsinki Declaration were followed in lieu of formal ethics committee approval and all study process was approved by the Gachon University Internal Review Board (Approval No. GCIRB 2017–296).

### Measures of depressive symptomatology

The Beck Depression Inventory (BDI) is the most popular 21-item self-reported questionnaire to assess the severity of depressive symptoms and is used to screen patients for depression, even in cases where symptomatology overlaps with neurovegetative symptoms [8]. The BDI score ranges from 0 to 63, with higher scores indicating more severe depressive symptoms. All of the participants completed the BDI questionnaire with the aid of a trained psychiatric nurse.

### Study design

The participants completed a BDI questionnaire one month before (Time 1, Baseline) treatment for prostate cancer (ADT or prostatectomy). The ADT group completed another BDI questionnaire six months after ADT initiation, while the operation group completed another BDI questionnaire six months after prostatectomy (Time 2, Endpoint). Further, the following demographic data, laboratory results, and questionnaire results (Variables-Baseline) were collected at Time 1: Age, BMI, IIEF, testosterone level, PSA level, Gleason score (GS), T stage. In addition, the following variables (variables-Change) that changed from Time 1 to Time 2 were measured: BMI, IIEF, testosterone level, and PSA level. The data collection interval was set to six months after treatment initiation as this is the point at which several experts examined the presence of side effects after surgical treatment of prostate cancer, and subsequent QoL in patients who underwent prostatectomy [9]. To identify the factors that affect the mean change in BDI score on ADT group, the confirmed variables were statistically analyzed.

### Propensity score analysis

We implemented an adjustment for differences in baseline characteristics between the ADT and operation group by using a weighted logistic regression model with stabilized weights. Stabilized weights have been proposed for modeling time-varying treatment status to reduce the potential for confounders that could cause selection bias in observational studies [10]. Propensity score analysis requires the formation of pairs from the ADT and operation groups with similar propensity score (PS) values. Hence, a logistic regression model was used to calculate and save the predicted probability of the dependent variable and the PS for each observation in the data set. This propensity score (between 0 and 1) represented the relationship between multiple characteristics and the dependent variable, as a single characteristic. Age, BMI, testosterone level, PSA level, IIEF, GS and T stage were used as independent variables, and treatment (ADT or prostatectomy) was used as the dependent variable for the propensity score model.

### Statistical analysis

Categorical variables were presented as numbers and percentages and were compared using the chi-square test. Continuous variables were presented as mean ± standard deviation and were compared using the t-test. To examine the BDI scores in the ADT and operation groups and to identify the variables that affect the BDI score, we analyzed distribution patterns before and after performing PS weighting (PSW). After PSW, we performed t-tests between ADT and operation groups to reduce the selection bias at baseline. We then compared the BDI score at Time 1 and Time 2, as well as changes in the values between the ADT and operation groups, using t-tests. Next, multivariate regression analysis was performed to identify the factors affecting depressive symptoms in the ADT group. The dependent variable was the change in BDI score, and the independent variables were age, BMI (baseline and change), testosterone level (baseline and change), PSA level (baseline and change), IIEF (baseline and change), and GS. Statistical analysis was performed using R version 3.5.1 (The R Foundation for Statistical Computing). All statistics were two-tailed and p-values <0.05 were considered to be significant.

## Results

**Fig 1:**
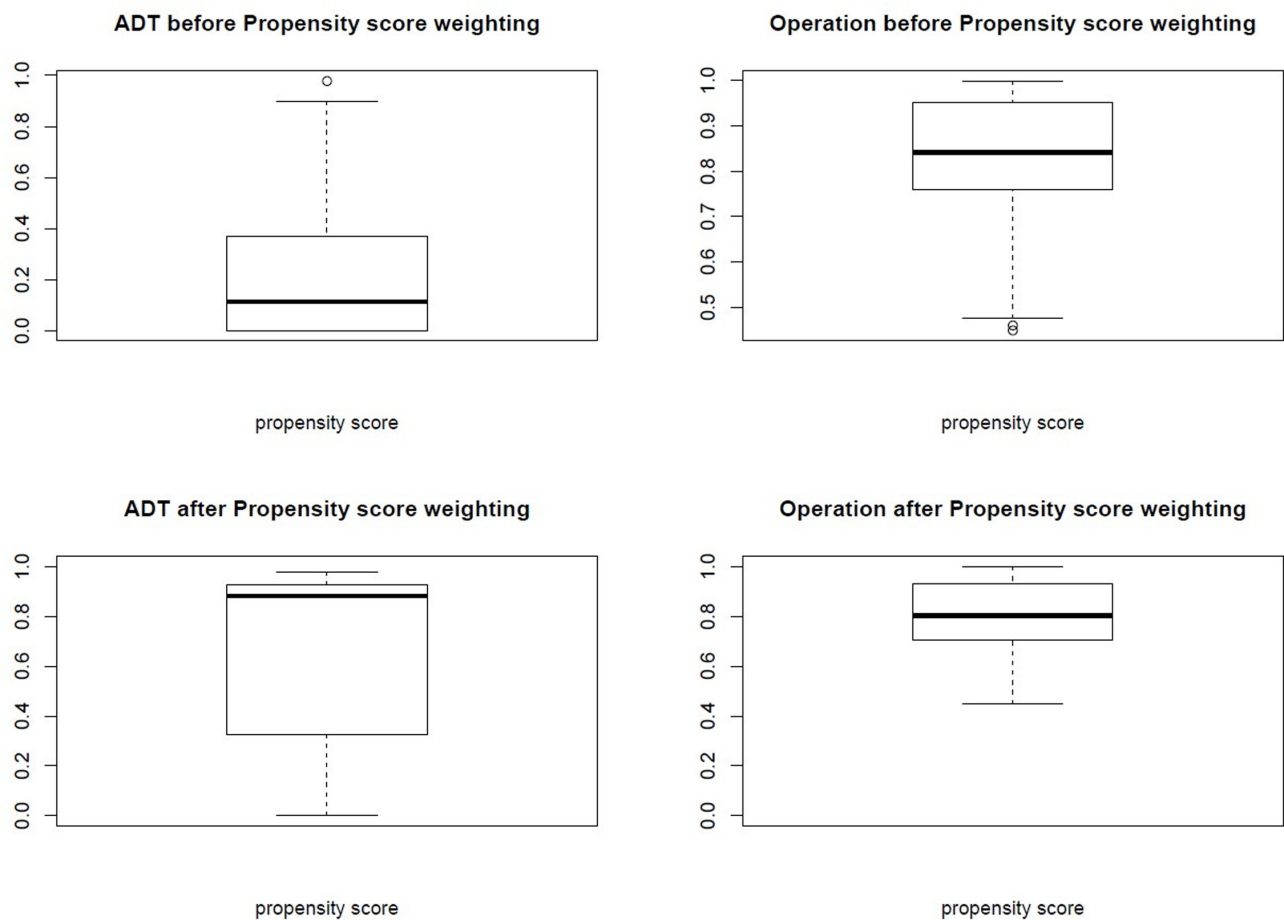
Comparison of differences in variables between the Androgen-Deprivation Therapy (ADT) group and the Operation group, using box plots, before and after propensity score weighting.

The distributions of participants before and after PSW are shown in Fig 1. Before PSW, the mean propensity scores were 0.24±0.32 in the ADT group and 0.83±0.15 in the operation group. The values were statistically significantly different between the two groups (*p* < 0.001), which indicates that there were only a small number of patients with similar variables between the two groups. However, after PSW, both groups had similar patient distributions, and the means and standard deviations at the baseline were well adjusted, with no statistically significant differences between the ADT group and the operation group (0.71±0.37 and 0.79±0.16, respectively; *p* = 0.493) **(Fig 1)**. After PSW, the number of participants in the pseudo population ADT group and operation group was 60.76 and 35.16, respectively, and none of the variables (age, BMI, testosterone level, PSA level, IIEF, GS, or T stage) was significantly different between the two groups at Time 1 **(Table 1)**.

**Table 1:** Baseline characteristics between the ADT and Operation groups before and after propensity score weighting.

Variables	Before propensity score weighting				After propensity score weighting		
	ADT Group	Operation Group	p-value		ADT Group	Operation Group	p-value
Patients number	49	58			60.76	35.16	
Age (year) ^a^	73.35 (7.62)	68.66 (6.07)		0.001	62.18 (10.57)	68.34 (5.94)	0.224
BMI (kg/m ^2^) ^a^	24.47 (2.91)	26.23 (2.41)		0.001	28.34 (3.89)	25.50 (1.99)	0.160
IIEF ^a^	4.36 (5.53)	4.38 (4.22)		0.982	4.68 (4.06)	4.49 (4.44)	0.855
Testosterone (ng/dL) ^a^	244.45 (256.63)	352.44 (186.90)		0.014	162.10 (247.99)	349.03 (180.98)	0.061
PSA (ng/ml) ^a^	77.51 (172.33)	11.78 (18.20)		0.011	26.82 (106.41)	12.09 (19.21)	0.344
Gleason score (%)				0.536			0.098
≦6	10 (22.2)	18 (31.0)			11.5 (21.9)	12.0 (32.9)	
7	19 (42.2)	24 (41.4)			8.8 (16.7)	15.2 (41.4)	
≧8	16 (35.6)	16 (27.6)			32.1 (61.3)	9.4 (25.7)	
T stage (%)				<0.001			0.062
T2	21 (43.2)	48 (83.3)			26.0 (42.8)	29.1 (83.6)	
T3	16 (31.8)	10 (16.7)			29.9 (49.2)	6.1 (16.4)	
T4	12 (25.0)	0 (0.0)			4.9 (8.0)	0.0 (0.0)	

Propensity score model: dependent variable (ADT vs Operation), independent variable (age, BMI, testosterone, PSA, IIEF, Gleasonscore). * Indicates statistically significant differences between categories using a chi-square test.

^a^ Values are presented as arithmetic mean (SD)

**Abbreviations:** ADT, Androgen-deprivation therapy; BMI, body mass index; IIEF, international index of erectile function; PSA, prostate-specific antigen.

### Changes in variables: BDI score, testosterone levels, IIEF, PSA, and T stage

After PSW, changes in the BDI score, testosterone level, PSA level, IIEF, and T stage at Time 2 were compared to the corresponding scores at Time 1 and these comparisons are detailed in Table 2. At Time 1, the mean BDI score was 10.27 in the ADT group and 10.74 in the operation group, with no statistically significant difference (*p* = 0.784). At Time 2, the scores were 19.40 and 8.98, respectively (*p* < 0.001), demonstrating a greater increase in the BDI score in the ADT group than in the operation group (ADT Group: 9.13, Operation group: -1.75) (*p* < 0.001). The mean testosterone level in the ADT and operation groups at Time 1 were 162.10 ng/dL and 349.03 ng/dL, respectively, while the mean IIEF scores were 4.68 and 4.49, respectively, neither of which were significantly different (*p* = 0.061, *p* = 0.855, respectively). At Time 2, the mean testosterone level was 30.83 ng/dL and 380.71 ng/dL, respectively, and the mean IIEF scores were 1.20 and 3.61, respectively (*p* < 0.001, *p* < 0.001). This shows that the ADT group had a greater reduction in testosterone (ADT group: -178.27, Operation group: 23.07) (*p* < 0.001) as well as in IIEF levels (ADT group: -3.48, Operation group: -0.88) (*p* = 0.008) compared to the operation group. The mean PSA levels in the ADT and operation groups were 26.82 and 12.09, respectively (*p* = 0.344) at Time 1, and 0.44 and 0.00, respectively (*p* = 0.352) at Time 2. There were no statistically significant differences in PSA levels between the ADT and operation groups at Time 1 or Time 2. In the T stage, there were also no statistically significant differences between groups (*p = 0*.*062*, respectively).

**Table 2:** The mean change in characteristics from the baseline value after propensity score weighting.

Variables	Time 1			Time 2				Mean change from baseline		
	ADT Group	Operation Group	p-value	ADT Group	Operation Group		p-value	ADT Group	Operation Group	p-value
BDI (Score) ^a^	10.27 (5.85)	10.74 (9.39)	0.784	19.40 (6.44)		8.98 (8.70)	<0.001	9.13 (4.63)	-1.75 (2.69)	<0.001
Testosterone (ng/dL) ^a^	162.10 (247.99)	349.03 (180.98)	0.061	30.83 (76.74)		380.71 (195.59)	<0.001	-178.27 (204.43)	27.07 (108.11)	<0.001
IIEF ^a^	4.68 (4.06)	4.49 (4.44)	0.855	1.20 (1.21)		3.61 (3.20)	<0.001	-3.48 (3.81)	-0.88 (3.54)	0.008
PSA (ng/ml) ^a^	26.82 (106.41)	12.09 (19.21)	0.344	0.44 (1.31)		0.00 (0.01)	0.352	-28.28 (111.49)	-12.09 (19.20)	0.352
T stage (%)			0.062				0.062	-	-	-
T2	26.0 (42.8)	29.1 (83.6)		26.0 (42.8)		29.1 (83.6)				
T3	29.9 (49.2)	6.1 (16.4)		29.9 (49.2)		6.1 (16.4)				
T4	4.9 (8.0)	0.0 (0.0)		4.9 (8.0)		0.0 (0.0)				

^a^ Values are presented as arithmetic mean (SD)

**Abbreviations:** BDI, Beck Depression Inventory; ADT, Androgen-deprivation therapy; IIEF, International index of erectile function; PSA, Prostate-specific antigen

### Variables affecting depressive symptoms

Multiple linear regression analyses were used to identify risk factors of depressive symptoms in the ADT group. At Time 1, with the mean change of BDI score as the dependent variable and age, BMI-baseline, testosterone level-baseline, PSA level-baseline, IIEF-baseline, and GS as the independent variables, the BDI score significantly increased by 0.446 according to IIEF-baseline (*p* = 0.019). (**Table 3; Model 1**) At Time 2, with the mean change in BDI score as the dependent variable and age, BMI change, testosterone level change, PSA level change, IIEF change, and GS as the independent variables, the BDI score significantly increased by 1.579 according to BMI change (*p* = 0.021) and decreased by 0.01 according to testosterone change (*p* = 0.034). (**Table 3; Model 2**)

**Table 3:** Multiple linear regression analysis of the baseline values and the mean change from the baseline after propensity score weighting.

	Model 1				Model 2		
	Coefficient	se	p-value		Coefficient	se	p-value
Age	-0.029	0.084	0.732	Age	-0.167	0.131	0.227
BMI-baseline	0.501	0.261	0.066	BMI change	1.579	0.597	0.021
Testosterone-baseline	-0.003	0.004	0.528	Testosterone change	-0.010	0.004	0.034
PSA-baseline	0.002	0.005	0.671	PSA change	-0.282	0.593	0.643
IIEF-baseline	0.446	0.179	0.019	IIEF change	-0.024	0.183	0.897
Gleason score				Gleason score			
≦6	Ref			≦6	Ref		
7	-1.575	2.119	0.463	7	-3.262	3.815	0.410
≧8	-3.256	2.348	0.1769	≧8	5.573	3.580	0.146

Model 1: dependent variable (mean change in BDI score), independent variables (age, BMI, testosterone-baseline, PSA-baseline, IIEF-baseline, Gleason score).

Model 2: dependent variable (mean change in BDI score), independent variables (age, BMI change, testosterone change, PSA change, IIEF change, Gleason score).

**Abbreviations:** se, Standard error; ADT, Androgen deprivation therapy; BMI, Body mass index; IIEF, International index of erectile function; PSA, Prostate-specific antigen.

## Discussion

In this study, we compared the depressive symptoms of patients undergoing ADT and prostatectomy as treatments for prostate cancer and found that patients undergoing ADT showed a worsening of depressive symptoms. In particular, patients undergoing ADT with a high IIEF score before ADT or patients undergoing ADT with reduced testosterone levels or increased BMI during ADT were likely to experience an exacerbation of depressive symptoms.

Management of survivorship issues is crucial for prostate cancer patients, and the key to patient management is to maintain a good QoL from diagnosis to after treatment [5]. Depression is the most common psychological problem affecting cancer patients [11]. In particular, prostate cancer is associated with the reproductive and urinary systems, so patients may feel shame and embarrassment following diagnosis. However, as cancer patients suffering from depressive symptoms are unlikely to seek professional help for their depressive symptoms, prostate cancer patients with exacerbated depressive symptoms require effective and timely intervention by healthcare professionals [12].

ADT is the prostate cancer treatment that has been most extensively studied in association with depression. Since the first report of an association between ADT and depressive symptoms in 1995, a variety of studies have been conducted to examine this relationship [13]. One study argued that ADT affects neurochemicals such as serotonin and thus has an adverse impact on depressive symptoms [14], while another study suggested that ADT affects pro-inflammatory cytokines such as IL-1 and IL-6 and thus worsens depressive symptoms [15]. Multiple studies have investigated the association between ADT and depressive symptoms using various control groups. In a study comparing patients undergoing ADT as a treatment for locally advanced prostate cancer and age-matched healthy men, depressive symptoms were significantly higher among those undergoing ADT [16]. However, a study comparing prostate cancer patients undergoing radiation found no significant correlation between ADT and depressive symptoms [17], and a study that compared prostate cancer patients who did not undergo ADT with patients on ADT could not confirm a correlation between ADT and exacerbation of depressive symptoms [18]. As shown here, the results of studies examining the association between ADT and depressive symptoms vary widely. In the present study, we compared depressive symptoms between patients undergoing ADT with those undergoing prostatectomy as the control group. After PSW, patients undergoing ADT, which did not significantly differ from those undergoing prostatectomy in terms of BDI scores before treatment, showed a significantly greater elevation of the BDI score during treatment than the patients undergoing prostatectomy (*p<0*.*001*). These results indicated that ADT is significantly correlated with depressive symptoms.

There have been several attempts to identify the risk factors of depressive symptoms during prostate cancer treatment [19]. Erectile dysfunction (ED), the most well-known side effect of ADT, has been found to be most closely related to depressive symptoms. For this reason, patients undergoing ADT experience delayed orgasm or an inability to attain orgasm, as well as reduced orgasmic intensity. The consequent decline in sexual interest undermines the emotional and physical intimacy with their spouse, inducing serious psychological distress [20,21]. This study found that patients with a high IIEF score before ADT are likely to experience an exacerbation of depressive symptoms during treatment (*p* = 0.019). This suggests that prostate cancer patients with higher EF before ADT may have an exacerbation of depressive symptoms during ADT. Therefore, clinicians treating prostate cancer need to monitor patients with high IIEF scores before ADT for depressive symptoms change during treatment.

Although the impact of ADT on depressive symptoms has not been elucidated, the most widely accepted notion is that a reduction in testosterone by ADT is the major cause of depressive symptoms [22]. A low testosterone level is related to depression, as testosterone reduction leads to a worsening of depressive symptoms, even in healthy men [22]. Additionally, a study that compared a group undergoing treatment with ADT for prostate cancer with several control groups showed that depressive symptoms increased as testosterone level decreased in the ADT group [23,24]. We found also that the testosterone reduction during ADT was significantly correlated with a worsening of depressive symptoms in patients treated with ADT (*p* = 0.034).

Previous studies have confirmed that changes in body mass composition beneficial for weight gain, such as a net decrease in bone density and lean muscle mass and an increase in fat mass, are an outcome of prostate cancer patients undergoing ADT [25]. According to a study that followed patients undergoing ADT for nine months, both whole body lean mass and bone mineral density decreased by a mean of 2.4%, while fat mass increased by a mean of 13.8% [26]. Moreover, patients undergoing ADT often show a decrease in physical activity and increase in fatigue levels. As a result, prostate cancer patients on ADT may exhibit an increase in weight and BMI, mostly within one year of treatment [27]. Further, multiple studies have confirmed that weight gain is significantly associated with depression. Obesity may induce depression over time through its negative effects on self-image or somatic consequences, and the risk for depressive symptoms is known to increase by 55% in obese people [28]. This study also found a significant correlation between increased BMI during ADT and exacerbation of depressive symptoms in patients undergoing ADT (*p* = 0.021).

This study found that depressive symptoms in prostate cancer patients undergoing ADT can be exacerbated and that high IIEF score before ADT, or reduced testosterone levels or increased BMI during ADT also have an impact on depressive symptoms. Based on these results, patients who are likely to become depressed during the course of ADT should be encouraged to complete a self-reported questionnaire or to regularly see a psychiatrist. Following confirmation of exacerbated depression, patients should be appropriately diagnosed and provided with treatment, such as antidepressant medications and psychotherapy.

This study has a few limitations. The sample size of this study was small, and a larger cohort should be used for future studies to improve the power of the data. We are planning to examine the relationships between various treatments for prostate cancer and changes in depressive symptoms as a follow-up study, and we hope this will address the limitations of this study.

## Conclusion

ADT is an essential and beneficial treatment for prostate cancer patients; however, it may exacerbate depressive symptoms. In particular, patients with a high IIEF before treatment, or patients who have a decline in testosterone level or increase BMI during treatment are highly likely to experience worsened depressive symptoms. This study highlights the correlation between ADT and depression and indicates the need for close monitoring of the mental wellbeing of prostate cancer patients to improve their QoL during and after treatment.

## Supporting information

S1 File. The Korean version of the Beck Depression Inventory. (PDF)

S2 File The Beck Depression Inventory. (PDF)
