# Supplementary material for: Changes in Beck Depression Inventory scores in prostate cancer patients undergoing androgen deprivation therapy or prostatectomy
Source: PLoS One. 2020 Jun 15;15(6):e0234264. doi: 10.1371/journal.pone.0234264 (PMC7295190; doi:10.1371/journal.pone.0234264)
Supplement: S1 File. The — (PDF) [file pone.0234264.s001.pdf]

벡 우울 척도(BDI: Beck Depression Inventory)

다음의 문항을 읽어보시고 각 번호의 여러 란 중에서 요즈음 자신에게 가장 적합하다고 생각되는 번호를 하나씩만 골라 ○표를 하십시오.

---

1. 1) 나는 슬프지 않다. ( )

   2) 나는 슬프다. ( )

   3) 나는 항상 슬프고 기운을 낼 수 없다. ( )

   4) 나는 너무나 슬프고 불행해서 도저히 견딜 수 없다. ( )

2. 1) 나는 앞날에 대해서 별로 낙심하지 않는다. ( )

   2) 나는 앞날에 대해서 용기가 나지 않는다. ( )

   3) 나는 앞날에 대해 기대할 것이 아무것도 없다고 느낀다. ( )

   4) 나는 앞날은 아주 절망적이고 나아질 가망이 없다고 느낀다. ( )

3. 1) 나는 실패자라고 느끼지 않는다. ( )

   2) 나는 보통사람들보다 더 많이 실패한 것 같다. ( )

   3) 내가 살아온 과거를 뒤돌아 보면, 실패투성이인 것 같다. ( )

4) 나는 인간으로서 완전한 실패자라고 느낀다. ( )

4. 1) 나는 전과같이 일상생활에 만족하고 있다. ( )

2) 나의 일상생활은 예전처럼 즐겁지 않다. ( )

3) 나는 요즘에는 어떤 것에서도 별로 만족을 얻지 못한다. ( )

4) 나는 모든 것이 다 불만스럽고 싫증난다. ( )

5. 1) 나는 특별히 죄책감을 느끼지 않는다. ( )

2) 나는 죄책감을 느낄 때가 많다. ( )

3) 나는 죄책감을 느낄때가 아주 많다. ( )

4) 나는 항상 죄책감에 시달리고 있다. ( )

6. 1) 나는 벌을 받고 있다고 느끼지 않는다. ( )

2) 나는 어쩌면 벌을 받을지도 모른다는 느낌이 든다. ( )

3) 나는 벌을 받을 것 같다. ( )

4) 나는 지금 벌을 받고 있다고 느낀다. ( )

7. 1) 나는 나 자신에게 실망하지 않는다. ( )

2) 나는 나 자신에게 실망하고 있다. ( )

3) 나는 나 자신에게 화가 난다. ( )

4) 나는 나 자신을 증오한다. ( )

8. 1) 내가 다른 사람보다 못한 것 같지는 않다. ( )

2) 나는 나의 약점이나 실수에 대해서 나 자신을 탓하는 편이다. ( )

3) 내가 한 일이 잘못되었을 때는 언제나 나를 탓한다. ( )

4) 일어나는 모든 나쁜 일들은 다 내 탓이다. ( )

9. 1) 나는 자살같은 것은 생각하지 않는다. ( )

2) 나는 자살할 생각을 가끔 하지만, 실제로 하지는 않을 것이다. ( )

3) 자살하고 싶은 생각이 자주 든다. ( )

4) 나는 기회만 있으면 자살하겠다. ( )

10. 1) 나는 평소보다 더 울지는 않는다. ( )

2) 나는 전보다 더 많이 운다. ( )

3) 나는 요즈음 항상 운다. ( )

4) 나는 전에는 울고 싶을 때 울 수 있었지만 요즈음은 울래야 울 기력조차

없다. ( )

11. 1) 나는 요즈음 평소보다 더 짜증을 내는 편은 아니다. ( )

2) 나는 요즈음 항상 짜증이 나고 귀찮아 진다. ( )

3) 나는 요즈음 항상 짜증을 내고 있다. ( )

4) 전에는 짜증스럽던 일에 요즘은 너무 지쳐서 짜증조차 나지 않는다. ( )

12. 1) 나는 다른 사람들에 대한 관심을 잃지 않고 있다. ( )

2) 나는 전보다 다른 사람들에 대한 관심이 줄었다. ( )

3) 나는 다른 사람들에 대한 관심이 거의 없어졌다. ( )

4) 나는 다른 사람들에 대한 관심이 완전히 없어졌다. ( )

13. 1) 나는 평소처럼 결정을 잘 내린다. ( )

2) 나는 결정을 미루는 때가 전보다 더 많다. ( )

3) 나는 전에 비해 결정 내리는 데에 더 큰 어려움을 느낀다. ( )

4) 나는 더 이상 아무 결정도 내릴 수가 없다. ( )

14. 1) 나는 전보다 내 모습이 나빠졌다고 느끼지 않는다. ( )

2) 나는 나이 들어 보이거나 매력 없이 보일 까봐 걱정한다. ( )

3) 나는 매 모습이 매력 없게 변해버린 것 같은 느낌이 든다. ( )

4) 나는 내가 추하게 보인다고 믿는다. ( )

15. 1) 나는 전처럼 일을 할 수 있다. ( )

2) 어떤 일을 시작하는 데에 전보다 더 많은 노력이 든다. ( )

3) 무슨 일이든 하려면 나 자신을 매우 심하게 채찍질해야만 한다. ( )

4) 나는 전혀 아무 일도 할 수가 없다. ( )

16. 1) 나는 평소처럼 잠을 잘 수 있다. ( )

2) 나는 전 만큼 잠을 자지는 못한다. ( )

3) 나는 전보다 한두 시간 일찍 깨고 다시 잠들기 어렵다. ( )

4) 나는 평소보다 몇 시간이나 일찍 깨고, 한번 깨면 다시 잠들 수 없다. ( )

17. 1) 나는 평소 보다 더 피곤하지는 않다. ( )

2) 나는 전보다 더 쉽게 피곤해 진다. ( )

3) 나는 무엇을 해도 피곤해진다. ( )

4) 나는 너무나 피곤해서 아무 일도 할 수 없다. ( )

18. 1) 내 식욕은 평소와 다름없다. ( )

2) 나는 요즈음 전보다 식욕이 좋지 않다. ( )

3) 나는 요즈음 식욕이 많이 떨어졌다. ( )

4) 요즈음에는 전혀 식욕이 없다. ( )

19. 1) 요즈음 체중이 별로 줄지 않았다. ( )

2) 전보다 몸무게가 2Kg가량 줄었다. ( )

3) 전보다 몸무게가 5Kg가량 줄었다. ( )

4) 전보다 몸무게가 7Kg가량 줄었다. ( )

*나는 현재 음식 조절로 체중을 줄이고 있는 중이다. (예, 아니오)

20. 1) 나는 건강에 대해 전보다 더 염려하고 있지는 않다. ( )

2) 나는 여러 가지 통증, 소화불량, 변비 등과 같은 신체적인 문제로 걱정하고 있다. ( )

3) 나는 건강이 염려되어 다른 일은 생각하기 힘들다. ( )

4) 나는 건강이 너무 염려되어 다른 일은 아무 것도 생각할 수 없다. ( )

21. 1) 나는 요즈음 성에 대한 관심에 별다른 변화가 있는 것 같지는 않다. ( )

2) 나는 전보다 성(sex)에 대한 관심이 줄었다. ( )

3) 나는 전보다 성(sex)에 대한 관심이 상당히 줄었다. ( )

4) 나는 성(sex)에 대한 관심을 완전히 잃었다. ( )

총점( )
