# Supplementary material for: Changes in Beck Depression Inventory scores in prostate cancer patients undergoing androgen deprivation therapy or prostatectomy
Source: PLoS One. 2020 Jun 15;15(6):e0234264. doi: 10.1371/journal.pone.0234264 (PMC7295190; doi:10.1371/journal.pone.0234264)
Supplement: S2 File — (PDF) [file pone.0234264.s002.pdf]

BECK DEPRESSION INVENTORY

Instructions: This questionnaire consists of 21 groups of statements. Please read each group of statements carefully and then pick out the one statement in each group that best describes the way you have been feeling during the past two weeks, including today. Circle the number beside the statement you have picked. If several statements in the group seem to apply equally well, circle the highest number for that group. Be sure that you do not choose more than one statement for any group, including Item 16 (Changes in Sleeping Patterns) or Item 18 (Changes in Appetite).

1.

0. I do not feel sad.

1. I feel sad most of the time.

2. I am sad all the time.

3. I am so sad or unhappy that I can't stand it.

2.

0. I am not discouraged about my future.

1. I feel more discouraged about my future than I used to.

2. I do not expect things to work out for me.

3. I feel my future is hopeless and will only get worse.

3.

0. I do not feel like a failure.

1. I have failed more than I should have.

2. As I look back, I see a lot of failures.

3. I feel I am a total failure as a person.

4.

0. I get as much pleasure as I ever did from the things I enjoy.

1. I don't enjoy things as much as I used to.

2. I get very little pleasure from the things I used to enjoy.

3. I can't get any pleasure from the things I used to enjoy.

5.

0. I don't feel particularly guilty.

1. I feel guilty over many things I have done or should have done.

2. I feel quite guilty most of the time.

3. I feel guilty all of the time.

6.

0. I don't feel I am being punished.

1. I feel I may be punished.

2. I expect to be punished.

3. I feel I am being punished.

7.

0. I feel the same about myself as ever.

1. I have lost confidence in myself.

2. I am disappointed in myself.

3. I dislike myself.

8.

0. I don't criticize or blame myself more than usual.

1. I am more critical of myself than I used to be.

2. I criticize myself for all of my faults.

3. I blame myself for everything bad that happens.

9. Suicidal Thoughts or Wishes

0. I don't have any thoughts of killing myself.

1. I have thoughts of killing myself, but I would not carry them out.

2. I would like to kill myself.

3. I would kill myself if I had the chance.

10.

0. I don't cry any more than I used to.

1. I cry more than I used to.

2. I cry over every little thing.

3. I feel like crying, but I can't.

11.

0. I am no more restless or wound up than usual.

1. I feel more restless or wound up than usual.

2. I am so restless or agitated that it's hard to stay still.

3. I am so restless or agitated that I have to keep moving or doing something.

12. Loss of Interest

0. I have not lost interest in other people or activities.

1. I am less interested in other people or things than before.

2. I have lost most of my interest in other people or things.

3. It's hard to get interested in anything.

13.

0. I make decisions about as well as ever.

1. I find it more difficult to make decisions than usual.

2. I have much greater difficulty in making decisions than I used to.

3. I have trouble making any decisions.

14.

0. I do not feel I am worthless.

1. I don't consider myself as worthwhile and useful as I used to.

2. I feel more worthless as compared to others.

3. I feel utterly worthless.

15.

0. I have as much energy as ever.

1. I have less energy than I used to have.

2. I don't have enough energy to do very much.

3. I don't have enough energy to do anything.

16.

0. I have not experienced any change in my sleeping patterns.

1a I sleep somewhat more than usual.

1b I sleep somewhat less than usual.

2a I sleep a lot more than usual.

2b I sleep a lot less than usual.

3a I sleep most of the day.

3b I wake up 1-2 hours early and can't get back to sleep.

17.

0. I am not more irritable than usual.

1. I am more irritable than usual.

2. I am much more irritable than usual.

3. I am irritable all the time.

18.

0. I have not experienced any change in my appetite.

1a My appetite is somewhat smaller than usual.

1b My appetite is somewhat greater than usual.

2a My appetite is much smaller than before.

2b My appetite is much greater than usual.

3a I have no appetite at all.

3b I crave food all the time.

19. Concentration Difficulty

0. I can concentrate as well as ever.

1. I can't concentrate as well as usual.

2. It's hard to keep my mind on anything for very long.

3. I find I can't concentrate on anything.

20.

0. I am no more tired or fatigued than usual.

1. I get more tired or fatigued more easily than usual.

2. I am too tired or fatigued to do a lot of the things I used to do.

3. I am too tired or fatigued to do most of the things I used to do.

21.

0. I have not noticed any recent change in my interest in sex.

1. I am less interested in sex than I used to be.

2. I am much less interested in sex now.

3. I have lost interest in sex completely.

Total Score: _______
